# Development and Characterization of *trans*-Cinnamaldehyde-Entrapped Zeolitic Imidazole Framework‑8 as an Antibacterial Agent for Food Safety Applications

**DOI:** 10.1021/acsomega.5c04494

**Published:** 2025-09-18

**Authors:** Zeynep Sevimli Yurttas, Rosana G. Moreira, Elena Castell-Perez

**Affiliations:** † Research Assistant, 14736 Texas A&M University, College Station, Texas 77843-2217, United States; ‡ Professor, Department of Biological and Agricultural Engineering, 14736 Texas A&M University, College Station, Texas 77843-2217, United States

## Abstract

This study shows successful synthesis, characterization, and demonstration of antibacterial activity of *trans*-cinnamaldehyde (TC)-loaded ZIF-8 nanoparticle complexes, highlighting their potential in the safety of food preparation surfaces and antimicrobial packaging, pH-responsive drug delivery surface cleaning, and biofilm prevention applications. The best ratio of TC to zinc + 2- was 1:2 in terms of a higher entrapment efficiency. The ZIF-8 nanoparticles were in the range of 100–200 nm and consistent with entrapment efficiency trends, while the poly-l-lysine (PL) coating increased the size beyond 300 nm. SEM and TEM images confirmed that TC entrapment did not change the morphology of the ZIF-8 nanoparticles. Gas adsorption analysis yielded a high BET surface area, which decreased upon TC entrapment. FTIR spectra exhibited distinctive peaks of ZIF-8 and TC in the nanoparticles, further confirming the successful synthesis procedure. Release studies indicated a burst release of TC in PBS, with water- and alcohol-based media enabling more gradual and sustained release.

## Graphical abstract

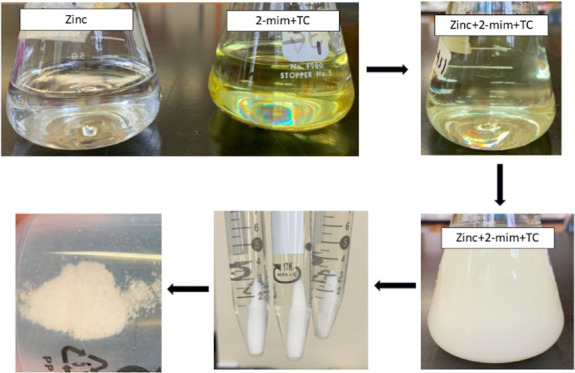

## Introduction

Foodborne illness is an avoidable public health concern that leads to approximately 48 million illnesses and 3000 deaths annually in the United States.^1^ These illnesses are often caused by harmful pathogens that can be introduced during the food production process or in commercial food settings, typically due to improper handling or cross-contamination. Therefore, proper manufacturing, testing, food handling, cleaning, and sanitation play a critical role.^2^ The most used disinfectants in the food industry are quaternary ammonium compounds (QAC), chlorine-based sanitizers, amphoteric compounds, hypochlorites, peroxides (peracetic acid and hydrogen peroxide), aldehydes (formaldehyde, glutaraldehyde, and paraformaldehyde), and phenolics.^3,4^ As a greener alternative, essential oils (EOs) are gaining attention. EOs are plant-derived secondary metabolites and have pharmacological properties such as anti-inflammatory, antioxidant, and anticarcinogenic effects, while some others act as biocides, targeting a wide range of organisms such as bacteria, fungi, viruses, protozoa, insects, and plants.^5^

*trans*-Cinnamaldehyde (TC) is a major component in *Cinnamomum cassia* spice, and it is the main reason for its antimicrobial properties.^6^ The acrolein group (α, β-unsaturated carbonyl moiety) in the structure of cinnamaldehyde is thought to be an essential element for antibacterial activity.^7^ TC is recognized as generally safe (GRAS) by both the United States Food and Drug Administration (FDA) and by the Flavor and Extract Manufacturers’ Association.^8^ The FDA and the Council of Europe recommend a daily intake limit of 1.25 mg/kg for TC.^9^ TC has been reported to prevent the growth of bacteria,^6,10,11^ molds,^12–14^ and yeasts.^15^ The antimicrobial activity of TC comes from its ability to interact with the bacterial cell wall and disrupt the cell wall structure.^16^ However, like other EOs, TC also has limitations on its practical applications due to hydrophobicity, low water solubility, intense aroma, and high reactivity.^17^ Nanoencapsulation offers a potential solution by enhancing its stability and improving its effectiveness.

Recently, metal–organic frameworks (MOFs) gained significant attention in drug delivery applications due to their high porosity and large surface area, ample pore volume for encapsulating substances, and strong chemical and thermal stability.^18^ Zeolitic imidazole framework-8 (ZIF-8) is a subgroup of MOFs with a zeolite solidate topology. ZIF-8 gained significant attention in biomedical applications, including drug delivery,^19–21^ tissue engineering,^22^ and antimicrobial therapy.^23–25^ The gentiopicroside^26^ (GPS) is a crystalline monoterpene secoiridoid glycoside and has an extremely bitter taste. The antibacterial activity of GPS-encapsulated ZIF-8 (GPS@ZIF-8) was tested via the agar well diffusion method against *Escherichia coli* (wild), *Pseudomonas putida*, and *Staphylococcus aureus,* and it was found that ZIF-8 encapsulation of GPS provided 2-fold increased antibacterial activity compared to pure GPS against the tested bacteria. The vancomycin and folic acid^27^ were coencapsulated in ZIF-8 and tested against multidrug-resistant *S. aureus* and exhibited similar zone inhibitions to a sensitive antibiotic used for the test as a positive control. Ciprofloxacin^28^ is another compound used to encapsulate into the ZIF-8 matrix. Its effectiveness against *E. coli* and *S. aureus* was tested via the disk diffusion method, and encapsulated particles showed almost 2-fold antibacterial activity compared to ciprofloxacin itself and almost 3-fold activity compared to only ZIF-8 nanoparticles.

While ZIF-8 materials have been widely reported for their antimicrobial activity, studies specifically of *trans*-cinnamaldehyde encapsulated in ZIF-8 remain limited. Our previous work^10^ primarily demonstrated the antibacterial efficacy of TC@ZIF-8 against *E. coli* O157:H7 on spinach leaves, focusing on its application in food safety. In contrast, the present study focuses on the comprehensive characterization of TC@ZIF-8 nanoparticles, including their particle size distribution, zeta potential, morphology (as observed by TEM and SEM), crystallinity (as determined by XRD), chemical composition and molecular structure (by FTIR spectroscopy), surface area (by BET), encapsulation efficiency, and controlled release behavior, as well as antimicrobial activity. These fundamental insights into the physicochemical properties of TC@ZIF-8 were not addressed in our earlier study and are crucial for advancing the understanding, optimization, and broader application of this system beyond food-surface disinfection.

The main goal of this study was to demonstrate that *trans*-cinnamaldehyde (TC)-loaded ZIF-8 nanoparticles can provide good, controlled release, maintain structural stability, and deliver effective antibacterial action, supporting their potential use in food safety, packaging, and other antimicrobial delivery applications. To that effect, we (1) evaluated and optimized the one-pot synthesis method for zeolitic imidazolate framework-8 (ZIF-8) nanoparticles to encapsulate the natural antimicrobial *trans*-cinnamaldehyde (TC); (2) investigated the TC release kinetics in different aqueous and organic media to determine how medium composition influenced release rates; and (3) evaluated the antibacterial efficacy of the ZIF-8 complexes against *E. coli* MG1655 under various conditions and compare their performance to free TC and the unmodified ZIF-8.

## Methodology

### Materials

Zinc nitrate hexahydrate (Zn (NO_3_)_2_·6H_2_O, 98%, Strem Chemicals, Newburyport, MA, USA), 2-methylimidazole (2-mim, C_4_H_6_N_2_, 99%, Acros Organics, Geel, Belgium), methanol (MeOH, BDH Chemicals, USA), ethanol (EtOH, EMPLURA, Supelco, Bellefonte, PA, USA), HCl acid (Acid Chlorhydrique, 34–37%, VWR Chemicals BDH, Radnor, PA, USA), acetonitrile (H3CCN, HPLC LC–MS grade, VWR, Radnor, PA, USA), Tween 20 (C58H114O26, Reagent grade, VWR, Radnor, PA, USA) potassium phosphate monobasic (KH_2_PO_4_, VWR Chemicals BDH, Radnor, PA, USA), potassium chloride (KCl, VWR Chemicals BDH, Radnor, PA, USA), sodium chloride (NaCl, VWR Chemicals BDH, Radnor, PA, USA), sodium hydroxide (NaOH, VWR Chemicals BDH, Radnor, PA, USA), and Petri dishes (VWR Disposable Sterile Petri Dishes, 100 × 15 mm, Radnor, PA, USA) were purchased from VWR (USA), and poly-l-lysine (PL) hydrobromide (Sigma-Aldrich, mol wt 1000–5000) was purchased from Krackeler Scientific, NY, USA. *trans*-Cinnamaldehyde (TC) (C_9_H_8_O, 99%, Acros Organics, Geel, Belgium), TSB (Difco Tryptic Soy Broth, Soybean-Casein Digest Medium, BD, Franklin Lakes, NJ, USA), TSA (Difco Tryptic Soy Agar, Soybean-Casein Digest Medium, BD, Franklin Lakes, NJ, USA), 96-well microtiter plates (Nunclon Delta Surface 96-wells, Thermo Scientific, Waltham, MA, USA), Mylar plate sealer (Thermo Scientific, Waltham, MA, USA), Buffered Peptone Water (CRITERION Dehydrated Culture Media, New York, NY, USA), and sodium phosphate (Dibasic, Anhydrous, Baker Chemical Co., Phillipsburg, NJ, USA) were purchased from Fisher Scientific (USA). All chemicals were used without further purification.

### Nanoparticle Synthesis

#### ZIF-8 Synthesis

ZIF-8 nanoparticles were synthesized using a method with slight modifications.^20^ Briefly, 0.94 g of zinc nitrate hexahydrate and 3.3 g of 2-methylimidazole were dissolved separately in a beaker containing 20 mL of absolute ethanol each and stirred for 15 min until they were completely soluble. The two beakers were placed in a water bath (30 °C) of an ultrasound instrument (510R-MT, Bransonic Ultrasonic Corp., Danbury, CT, USA; ultrasound frequency, 42 kHz; power consumption, total 70 W) for 5 min. The 2-methylimidazole ligand solution was then quickly added to the zinc nitrate solution, and the ultrasound was turned on for 60 min. The milky solution was divided into 10 mL in 15 mL centrifuge tubes, centrifuged at 10,000 rpm (Allegra TM 25R Centrifuge, Beckman Coulter, Brea, CA, USA) for 5 min, and washed thrice with 10 mL of EtOH after each step. After being washed, the collected ZIF-8 nanoparticles were dried in a vacuum oven equipped with a vacuum pump (Welch 1376 DuoSeal Vacuum Pump, Thomas Industries Inc., Skokie, IL, USA) at 35 °C for 2 h. The resultant ZIF-8 nanoparticles were placed in a desiccator and stored at room temperature for future testing and used within 2 weeks of synthesis.

#### Synthesis of TC@ZIF-8 Nanoparticle Synthesis and Coating with Poly-l-lysine

Encapsulation of TC in the ZIF-8 nanoparticles (Figure S1) was performed by the same one-pot synthesis method, with a slight modification in which a given amount of TC was added together with 2-methylimidazole when preparing the solution of the organic ligand.^10,20^ Different mass ratios of TC per total solid component were evaluated to find the relationship between the TC amount and total solid content (zinc nitrate hexahydrate + 2-mim). Similar to the ZIF-8 synthesis, approximately 0.94 g of zinc nitrate hexahydrate and 3.3 g of 2-methylimidazole were dissolved separately in an Erlenmeyer flask containing 20 mL of ethanol and stirred for 15 min until completely dissolved. TC was added to the 2-mim solution during the preparation. The two Erlenmeyer flasks were placed in a water bath (30 °C) of an ultrasound instrument (510R-MT, Bransonic Ultrasonic Corp., Danbury, CT, USA; ultrasound frequency, 42 kHz; power consumption, total 70 W) for 5 min. The 2-mim ligand solution containing TC was then quickly added to the zinc nitrate solution and sonicated for 60 min. The milky solution was centrifuged at 10,000 rpm for 5 min and washed thrice with 40 mL of EtOH each time. After washing, the ZIF-8/TC sediments were vacuum oven-dried with a pump (Welch 1376 DuoSeal Vacuum Pump, Thomas Industries Inc., Skokie, IL, USA) at 35 °C for 2 h. The resultant nanoparticles were placed in a desiccator and stored at room temperature for future testing, within 2 weeks.

To prevent aggregation and sedimentation, ZIF-8 and TC-encapsulated ZIF-8 nanoparticles were coated with PL.^29^ For the coating, ZIF-8 and TC@ZIF-8 nanoparticles were suspended in 0.1 mg/mL PL at a ratio of 1:1 (mg of ZIF-8: mL of 0.1 mg/mL PL), vortexed until fully dissolved, and sonicated in an ultrasound bath at 42 kHz ±6% and 70 W power consumption for 5 min. Then, ZIF-8-PL solutions were frozen overnight at −20 °C and then freeze-dried (FreeZone, Labconco Corp., Kansas City, MO, USA) at −50 °C and 0.120 mbar for 48 h. The PL-coated ZIF-8 (ZIF-8@PL or TC@ZIF-8@PL) particles were placed in a desiccator and stored at room temperature for future testing.

### Nanoparticle Characterization

#### Particle Size, Zeta Potential, and TC Entrapment Efficiency

The particle size was determined via nanoparticle tracking analysis (NTA) with a NanoSight NS 300 instrument (NanoSight, Malvern Panalytical Inc., Massachusetts, USA) equipped with a sample chamber with a 532 nm green laser and flow-cell top plate. Particles in a liquid suspension were loaded into a sample chamber illuminated by a specially shaped laser beam. The ZIF-8 and TC@ZIF-8 nanoparticles were dispersed in ethanol (the particle concentration is around 0.2 mg/mL) and injected into the sample chamber with a syringe (1 cc) connected with an infusion pump. The samples were recorded for 45 s, and 4 measurements were performed for each sample. The mean size, D10, D50, D90, and SD values obtained by the NTA software correspond to the arithmetic values calculated with the sizes of all the particles analyzed by the software. D90 values were reported for particle size. All of the measurements were performed at room temperature.

Zeta potential was measured with a Malvern Zeta Sizer (Malvern Zetasizer Nano ZS, Malvern Panalytical Inc., MA, USA) with a folded capillary zeta cell (DTS1070, Malvern Panalytical Inc., MA, USA). The zeta potential readings were carried out using freshly prepared ZIF-8 and TC@ZIF-8 suspensions in PL and after freeze-drying and resuspending in water. Each suspension (concentration, 2 mg/mL) was sonicated for 5 min prior to measurement.

The amount (percentage) of entrapped TC in the ZIF-8 (TC@ZIF-8) nanoparticles was determined by UV–visible spectrophotometry (Genesys 10S UV–vis Spectrophotometer, Thermo Fisher Scientific, Madison, WI, USA). Approximately 5 mg of TC@ZIF-8 was dissolved in 10 mL of 0.1 M HCl as sample solutions (diluted from hydrochloric acid, Acid Chlorhydrique, 34–37%, VWR Chemicals BDH, Radnor, PA, USA) at a concentration of 0.5 mg/mL and mixed thoroughly. The suspensions were left for 15 min under constant agitation and then placed in a UV–visible spectrophotometer, and the absorbance was measured at 295 nm. The wavelength was determined as the maximum absorbance in preliminary tests by scanning the UV–vis spectra of TC dissolved in 0.1 M HCI in triplicate. Samples were diluted with 0.1 M HCl for proper spectrophotometric readings to keep the absorbances within a linear range.

Entrapment efficiency (EE %) of the ZIF-8 nanoparticles was determined as^11^

1 $$EE\;\left(\%\right)=\frac{amount\;of\;active\;compound\;entrapped}{initial\;active\;compound\;amount}\times 100$$

where the “amount of active compound entrapped” is the amount of compound (TC in this study) present in the ZIF-8 particles in (mg/mL)/(mg/mL), and the “initial active compound amount” indicates the amount of compound (TC) initially used to manufacture the particles (g/g). A calibration curve was obtained for TC dissolved in the 0.1 M HCl solution, ranging from 0.5 to 6.0 μg/mL at 295 nm.

The entrapment efficiency (EE %) of the TC@ZIF-8 nanoparticles was measured because different mass ratios of TC per total solid component were used to synthesize the nanoparticles.

#### Scanning Electron Microscopy and Transmission Electron Microscopy

The morphology of ZIF-8, TC@ZIF-8, and TC@ZIF-8@PL nanoparticles was imaged using a JEOL JSM-7500F (JSM7500, RRID/SCR_022202, Materials Characterization Facility, Texas A&M University) ultrahigh-resolution field emission scanning electron microscope (FE-SEM) equipped with a high-brightness conical FE gun and a low-aberration conical objective lens. The nanoparticles (in powder form) were placed as a very thin layer on carbon tape and then sputter-coated (Cressington Sputter Coater, 208 HR, Ted Pella, Inc., CA, USA) with platinum/palladium with a thickness of 5 nm prior to imaging. SEM was operated at an accelerating voltage of 3 kV and a working distance between 8 and 15 mm, and the images were taken at a magnification of 37k ×.

Aqueous suspensions of ZIF-8, TC@ZIF-8, and TC@ZIF-8@PL were also examined using a FEI Morgagni Transmission Electron Microscope (FEI Company, Hillsboro, OR) (Image Analysis Lab at Texas A&M University). The suspensions of nanoparticles were placed on 300 mesh copper grids and stained with a 2% (w/v) uranyl acetate aqueous stain (Electron Microscopy Sciences, Hatfield, PA) to provide contrast under magnification. Excess liquid on the mesh was removed with filter paper, and the grid was allowed to dry before viewing under 50,000–100,000 times magnification. Observations were performed at 80 kV.

#### X-ray Diffraction Analysis, Gas Adsorption Analysis, and Fourier Transform Infrared Spectroscopy

X-ray diffraction analysis was conducted by using a Xeuss 2.0 HR SAXS/WAXS system (Xenocs, Sassenage, France) with a Cu source tuned to λ = 0.1542 nm. Measurements were conducted at sample to detector distances of 1455.25 and 156.75 mm.

Nitrogen gas adsorption experiments of ZIF-8, ZIF-8@PL, TC@ZIF-8, and TC@ZIF-8@PL were measured at 77 K on a Belsorp Max X adsorption analyzer at the Microtrac Applications Laboratory (Philadelphia, Pennsylvania, USA). Samples were pretreated at 100 °C under dynamic vacuum for 12 h before analysis. The 0.5 TC samples were pretreated at 70 °C under dynamic vacuum for 12 h. Brunauer–Emmett–Teller (BET) surface areas were calculated according to the ISO 9277 standard with Rouquerol fits. Pore size distributions were calculated according to the Barrett–Joyner–Halenda (BJH) method, and micropore volumes were determined via T-Plots.

FTIR spectroscopy was performed with a Nicolet iS50 FTIR spectrometer (Thermo Fisher Scientific, Madison, WI, USA) with an attenuated total reflectance (ATR) instrument. Prior to the sample examination, air (no samples in the sample holder) and water were used for background analysis. ZIF-8, ZIF-8@PL, TC@ZIF-8, and TC@ZIF-8@PL were used as synthesized powders, and *trans*-cinnamaldehyde was used as a liquid. The spectra were acquired using an XT-KBr beam splitter and DTGS ATR detector in the region of 4000–400 cm^–1^ wavenumbers, averaged from 64 scans with a resolution of 8 cm^–1^. The light source’s optical velocity for acquiring the spectra was 0.4747 with an aperture of 100 for sensitive detection and energy saturation prevention. The OMNICTM spectra program (Thermo Fisher Scientific, Madison, WI, USA), version 9.9.549 (2018), was used to gather and evaluate the spectrum.^30^

#### TC Release from ZIF-8

The release of TC from ZIF-8 nanoparticles and PL-coated particles was evaluated using PBS, pH 7.4, water, ethanol, methanol, a PBS/ethanol mixture (v/v, 20%), and a PBS/methanol mixture (v/v, 3%, 5%, 10%) as release mediums. A typical release system was prepared by suspending TC@ZIF-8 and TC@ZIF-8@PL nanoparticles in buffer media at a concentration of 1 mg/mL, and each suspension was prepared separately at each specific time interval and incubated in a water bath shaker (VWR International 89032-226, West Chester, PA) at 37 °C, 100 rpm for 5 days. At predetermined time intervals, a 1 mL aliquot was drawn and filtered through 0.2 μm nylon membrane syringe filters (VWR Intl., West Chester, PA) prior to measurement. The measurements were performed with UV–visible spectrophotometry (Genesys 10S UV–vis Spectrophotometer, Thermo Fisher Scientific, Madison, WI, USA). For each buffer, the wavelength was determined as the maximum absorbance in preliminary tests by scanning the UV–vis spectra of TC dissolved in each buffer. The absorbance readings were performed at 295 nm for water, PBS pH 7.4, and PBS: ethanol mixture and at 290 nm for methanol and ethanol. The same calibration curve obtained for encapsulation efficiency was used for water, PBS pH 7.4, and PBS: ethanol mixture, and a new calibration curve was obtained for ethanol and methanol by dissolving TC at a concentration between 0.5 and 6 μg/mL in ethanol and methanol.

### Antibacterial Activity

#### Bacterial Culture Preparation

*E. coli* MG1655 was obtained from the Texas A&M University Microbiology Laboratory culture collection (Department of Biological and Agricultural Engineering, College Station, TX, USA). The stock cultures were maintained at −80 °C in a 20% glycerol solution. Prior to use, the frozen culture was activated in trypticase soy broth (TSB) by transferring and incubating it aerobically for 24 h at 37 °C in an incubator (Symphony Forced Air General Incubator, VWR, Radnor, PA, USA). From this, the T strike method was used to plate on tryptic soy agar (TSA) for isolating colonies by identical duplicate transfers. Then, a single colony was transferred to the TSA slant and incubated for 24 h at 37 °C; then, the slant was stored in a refrigerator at 4 °C for future use (no more than three months). *E. coli* MG1655 was maintained, revived, and handled under biosafety level (BSL) 2 containment at all times according to Texas A&M University System Institutional Biosafety Committee (IBC) policy, and operations were carried out in a fume hood (Purifier Biological Safety Cabinet, Labconco Corporation, Kansas City, MO, USA). All the tips, media, containers, and utensils were sterilized in a high-pressure steam sterilizer (MLS-3781L Labo Autoclave, Sanyo Electric Co., Ltd., Osaka, Japan).

#### Minimum Inhibitory Concentration and Minimum Bactericidal Concentration

The MIC of ZIF-8 and TC@ZIF-8 nanoparticles, both with and without 0.1 mg/mL polylysine coating, was determined using a broth dilution assay.^31^ Bacterial cultures were incubated at 37 °C for 20–22 h in TSB, then centrifuged at 4000 rpm for 15 min, washed with sterile 0.1% peptone water (w/v) 3 times at room temperature, and then resuspended in TSB, and the optical density (OD) was measured at 630 nm. Using the equation below (OD to CFU/mL), the concentration was diluted with double-strength TSB (2× TSB) for an initial inoculum of approximately 5.0 log 10 CFU/mL in each sample well.

2 $$y=(1\times 10^{9})x-(6\times 10^{7})$$

where *x* is the absorbance of bacteria at 630 nm and *y* is the CFU/mL. Confirmation of CFU/mL was done by enumeration of the inoculum, which was completed by serial dilution in sterile 0.1% peptone water (w/v) and spread-plating it on Petri dishes containing TSA. The aliquots of 100 μL of all antimicrobial solutions and solvent blanks were spread-plated to ensure sterility, and all plates were incubated for 24 h at 37 °C. For MIC, the ZIF-8, ZIF-8@PL, TC@ZIF-8, and TC@ZIF-8@PL nanoparticle solutions were added to the microtiter plates as aqueous suspensions of different concentrations ranging from 1250 to 10,000 μg/mL for *E. coli* MG1655.^10^ TC was added as an aqueous solution containing 1.0 g/100 g acetonitrile and 0.01 g/100 g Tween 20 with concentrations ranging from 62.5 to 2000 μg/mL;^32^ PL was added in the range of 0.1–0.5 mg/mL. Equivalent volumes (100 μL) of the ZIF-8, ZIF-8@PL, TC@ZIF-8, and TC@ZIF-8@PL nanoparticle solutions and bacterial inoculum in 2× broth were loaded into each test well, denoted as the sample group (SPL) in 96-well microtiter plates. Sample controls (SPLC) consisted of 100 μL of nanoparticle solutions and 100 μL of 2× TSB to cancel the solution background color. Negative controls (NEG) were prepared with 100 μL of sterilized water and 100 μL of 2× TSB to account for baseline OD630 readings. Positive controls (POS) were prepared with 100 μL of the inoculum in 2× TSB and 100 μL of sterilized water to ensure that the liquid that dilutes the particles has no inhibitory effect on bacterial growth. SPL and SPLC groups were added at the initial concentration of antimicrobial solution to the first column and then serially diluted with 100 μL of sterilized distilled water. The final concentration of the bacterial inoculum was 5 × 10^5^ CFU/mL in the wells. Once the plate was prepared, it was covered with a Mylar plate sealer, incubated at 37 °C, and shaken gently in an ELISA reader (Synergy H1Microplate Reader, BioTek, Winooski, VT, USA) before each reading. The optical density (OD) at 630 nm was recorded every hour for 24 h in Gen 5 Microplate Plate and Imager Software Version 3.00 (BioTek, Winooski, VT, USA). Then, all the OD630 (SPL) values were normalized by using the OD630 (BLANK) values of the sample control with the equations below:^33^

3 $$\left(0_{h}OD_{630,SPL}\right)-\left(0_{h}OD_{630,SPLC}\right)=0_{h}OD_{630,normalized}$$

4 $$\left(24_{h}OD_{630,SPL}\right)-\left(24_{h}OD_{630,SPLC}\right)=24_{h}OD_{630,normalized}$$

5 $$\left(24_{h}OD_{630,normalized}\right)-\left(0_{h}OD_{630,normalized}\right)=\Delta OD_{630}$$

Any antimicrobial sample well that showed ≤ OD_NEG_ was effectively inhibited by the antimicrobial, with no growth of *E. coli* MG1655. The MICs were determined as the lowest concentration of an antimicrobial that inhibited growth for all test replicates for all microorganisms, respectively^31^

After the MIC test, all wells that showed inhibition of the test microorganism after 24 h were then tested for bactericidal activity by spreading 100 μL from each well showing inhibition onto TSA plates and incubating for 24 h at 37 °C. The lowest concentrations that showed 3-log reduction on the plate surfaces following incubation were considered bactericidal (MBC).^33^

### Statistical Analysis

All experiments were performed in triplicate as independent experiments unless noted otherwise. The data was expressed as mean values ± the standard deviation (SD). Statistical analysis was performed using SPSS Software (IBM SPSS Inc., version 29.0.0.0, Armonk, New York, USA). Statistical differences between variables were analyzed for significance by one-way analysis of variance (ANOVA) using Tukey’s multiple range tests, and if needed, Student’s *t*-test was performed to compare two independent means. Statistical significance was expressed at the *P* < 0.05 level.

## Results and Discussion

### Nanoparticle Characterization

#### Particle Size, Zeta Potential, and Entrapment Efficiency

Table 1 shows the size (D90 values) of TC-entrapped nanoparticles and PL-coated (samples denoted as @PL) nanoparticles. The size of TC-entrapped ZIF-8 nanoparticles increased with increasing TC amount; the change in size was significantly (*p* > 0.05) higher for 0.5TC@ZIF-8 nanoparticles compared to the ZIF-8s. The particle size was 113.4 nm, 145.8 nm, 171.9 nm, 213.6 nm, and 181.8 nm for ZIF-8, 0.1TC@ZIF-8, 0.25TC@ZIF-8, 0.5TC@ZIF-8, and 1TC@ZIF-8, respectively. On the other hand, PL-coated ZIF-8 (ZIF-8@PL) and 0.5TC@ZIF-8 (0.5TC@ZIF-8@PL) structures had higher particle sizes (*p* < 0.05) than the uncoated counterparts, 293 nm and 337 nm, respectively.

**Table 1:** Size (D90) of TC@ZIF-8 and PL-Coated (TC@ZIF-8@PL) Nanoparticles

^b^TC@ZIF-8	size, nm^a^	PL-coated	size, nm^a^
ZIF-8	113.4 ± 56.2^a^	ZIF-8	113.4 ± 56.2^a^
0.10TC@ZIF-8	145.8 ± 24.2^a,b^	Zif-8@PL	293.0 ± 61.3^b^
0.25TC@ZIF-8	171.9 ± 38.1^a,b^	0.5TC@ZIF-8	213.6 ± 74.7^a,b^
0.50TC@ZIF-8	213.6 ± 74.7^b^	0.5TC@ZIF-8@PL	337 ± 94.7^b^
1.00TC@ZIF-8	181.8 ± 11^a,b^		

a Values given are averages of four replicate sample size D90 values± standard deviations. ^ab^Means within a column, which are not followed by a common superscript letter, are significantly different (*p* < 0.05).

b Values before TC represent TC/(zinc + 2-mim) ratios (w/w).

#### Particle Size Measured with Nanoparticle Tracking Analysis

Figure 1 shows a narrow distribution for 0.1TC@ZIF-8 nanoparticles, while the others had wider size distributions.

**Figure 1:**
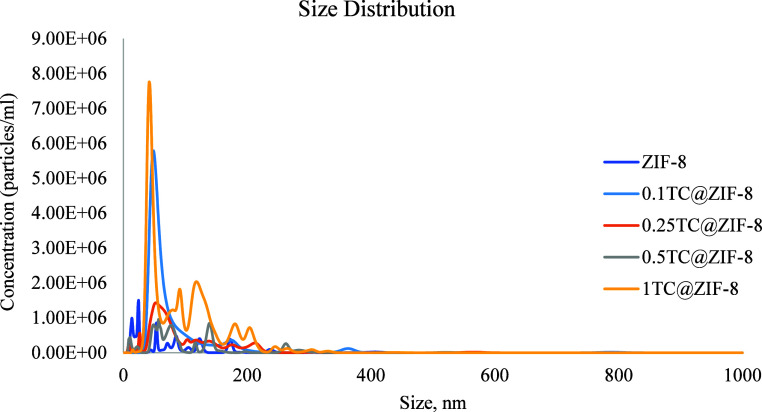
Size distribution of ZIF-8 and TC@ZIF-8 nanoparticles.

In this study, ethanol was used as a solvent, the ultrasound bath operated at 42 kHz, and the size of ZIF-8 was 113.4 nm. The impact of various experimental conditions, including stirring method, reagent concentration, solvent, and reaction time, was studied.^20^ The authors found that, compared to the magnetic stirring, the ultrasound method yielded smaller particle sizes (330 vs 480 nm) in a shorter reaction time (1 h) when operating at a 37 kHz frequency. ZIF-8 was synthesized using various methods, including solvothermal, microwave-assisted, sonochemical, mechanical, dry-gel, and microfluidic methods, and the physicochemical properties of the ZIF-8s were compared.^34^ This study used DMF and triethylamine (TEA) during the synthesis; the ultrasound instrument was operated at 20 kHz, and the reaction time was 1h, and ZIF-8 had a 300–500 nm size. These findings suggest that ZIF-8 particle size is influenced by the synthesis method, solvent choice, reaction time, and, notably, the frequency of the ultrasound used.

Table 2 shows the zeta potential values of TC-entrapped ZIF-8 nanoparticles and when coated with PL TC-entrapped ZIF-8 nanoparticles. In a preliminary study (data not shown), EE % values of 0.43TC@ZIF-8 and 0.67TC@ZIF-8 were close but still less than the EE % for the 0.5TC@ZIF-8 sample (32.4%). Considering this information, TC entrapment significantly lowered the zeta potential in the maximum EE % sample (0.5TC@ZIF-8) compared to the ZIF-8 sample. When coated with PL, the zeta potential increased (*p* < 0.05) significantly for all coated samples.

**Table 2:** Zeta Potential Values of TC-Entrapped (TC@ZIF-8) ZIF-8 and TC-Entrapped and PL-Coated ZIF-8 (TC@ZIF-8@PL) Nanoparticles

	ZP, mV^a^		ZP, mV^a^
ZIF-8	*x*36.25 ± 0.79^b^	ZIF-8@PL	*y*47.93 ± 1.40^a^
0.43TC@ZIF-8	*x*35.37 ± 0.58^a,b^	0.43TC@ZIF-8@PL	*y*51.43 ± 0.06^a,b^
0.5TC@ZIF-8	*x*33.033 ± 0.71^a^	0.5TC@ZIF-8@PL	*y*57.57 ± 2.97^a,c^
0.67TC@ZIF-8	*x*35.07 ± 0.71^a,b^	0.67TC@ZIF-8@PL	*y*54.17 ± 1.33^b,c^
1TC@ZIF-8	*x*37.23 ± 2.55^b^	1TC@ZIF-8@PL	*y*58.80 ± 0.92^c^

a Values are averages of three replicate samples ± standard deviations. ^a,b^Means within a column, which are not followed by a common superscript letter, are significantly different (*p* < 0.05). ^*x,y*^Means within a row, which are not followed by a common subscript letter, are significantly different (*p* < 0.05).

Zeta potentials indicate the surface charge of the nanoparticles and their stability. Both the particle size and zeta potential play important roles in drug delivery applications. Particle size not only shows the size of the particles but also gives information about the size distribution of the nanoparticles, while the zeta potential determines their stability in a system. For a stable nanoparticle suspension, the zeta potential ideally should be more than ±30 mV.^35^ However, the studies on the stability of ZIF-8 ^29^ showed that ZIF-8 sedimented in water even though the zeta potential was 36.25 mV. Commercial ZIF-8 powder has a zeta potential of 32.6 mV.^36^

The TC values (0.10, 0.25, 0.50, 0.75, and 1.00) in Table 3 represent the TC/(zinc + 2-mim) ratios (w/w). The entrapment efficiency increased (*p* < 0.05) as the TC/zinc+2-mim ratio increased, reaching a peak of 32.49% at a 1:2 ratio (Table 3). Beyond this point, even though the TC amount increased, the EE started to decrease (*p* < 0.05), likely due to the increased number of nucleation sites at high TC concentrations that resulted in insufficient encapsulation.^37^ The increase and then decrease in the entrapment efficiency (EE) of TC@ZIF-8 nanoparticles correlate with the nanoparticle size values. A similar trend was seen in a study where horseradish peroxide was encapsulated in ZIF-8.^37^ In that study, the encapsulation efficiency improved from around 28% to about 98% as the 2-mim to Zn^2+^ ratio decreased from 4:1 to 1:1. In this study, the concentration ratio of 2-mim to zinc nitrate was 3.5, and the maximum entrapment efficiency was 32%, closely aligning with the ∼34% efficiency reported at a similar ratio in the horseradish peroxide study.

**Table 3:** TC Entrapment Efficiency (%) Values for TC@ZIF-8 Nanoparticles for Different TC Ratios

^b^TC@ZIF-8	Entrapment Efficiency [%]^a^
0.10TC@ZIF-8	12.92 ± 0.58^a^
0.25TC@ZIF-8	16.59 ± 1.07^b^
0.50TC@ZIF-8	32.49 ± 0.26^e^
0.75TC@ZIF-8	29.15 ± 0.10^d^
1.00TC@ZIF-8	21.18 ± 0.65^c^

a Values given are averages of three replicate samples ± standard deviations. ^a,b,c,d,e^Means within a column, which are not followed by a common superscript letter, are significantly different (*p* < 0.05).

b Values before TC represents TC/(zinc + 2-mim) ratios (w/w).

Caffeine was encapsulated in a ZIF-8 matrix using two different approaches: a one-step method with 2 h of mixing and an impregnation method where already synthesized ZIF-8 was mixed with an aqueous caffeine solution at 80 °C for 1.5–8 days.^38^ It was found that the encapsulation efficiency (%) was 28.1% with the one-step method, while it required about 3 days to achieve similar results with the impregnation method. Curcumin was encapsulated into the ZIF-8 matrix using the one-step method, and it was found that the highest encapsulation efficiency occurred just after 15 min of mixing.^39^ These findings highlight that the encapsulation efficiency depends on several factors such as reaction time, temperature, entrapped drug, ratio of 2-mim to Zn^2+^, and method of encapsulation.

The nanoparticles with a mass ratio that yielded the highest entrapment efficiency were 0.5TC@ZIF-8 and were used for the remaining characterization methods.

#### SEM and TEM

SEM images (Figure 2) show typical hexagonal ZIF-8 formation in both ZIF-8 samples and *trans-*cinnamaldehyde-entrapped samples.

**Figure 2:**
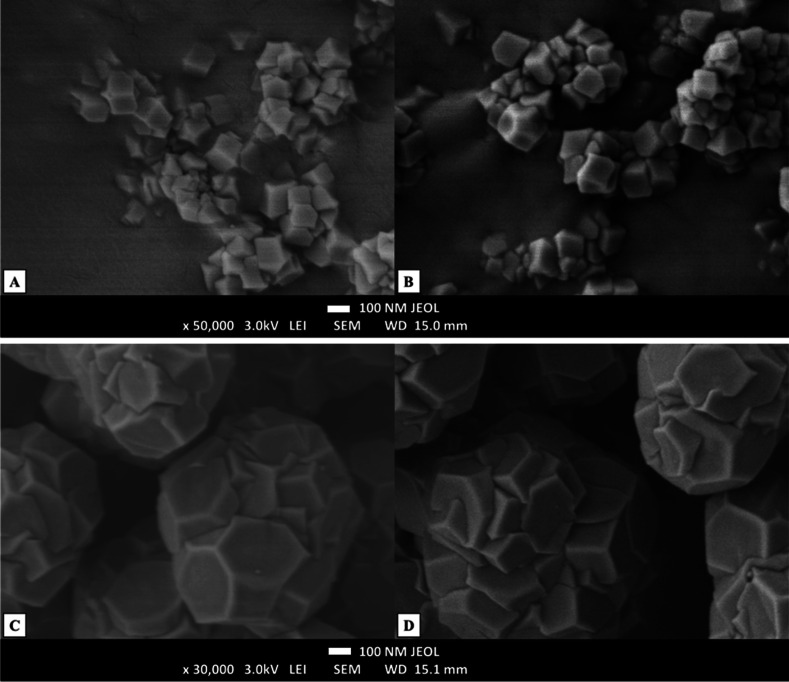
SEM images of (A) ZIF-8, (B) ZIF-8@PL, (C) 0.5TC@ZIF-8, and (D) 0.5TC@ZIF-8@PL.

ZIF-8 and ZIF-8@PL images (Figure 2A,B) appear as if they are hollow. ZIF-8 has been reported to have a pore size of 11.6 Å and window openings of 3.4 Å; however, it is observed that its structure allows entry of larger molecules than its window size.^40^

The ethanol molecules are approximately 4.5 Å in size,^41^ which is close to the aperture of the ZIF-8 cage. This allows them to be trapped within the pores and exit the structure during the drying process without displacing the ZIF-8 framework, although some structural defects may be introduced.^42^ However, after TC entrapment (Figure 2C,D), the frame appears more defined. This indicates that TC filled the pores inside the framework without any changes in the morphology of ZIF-8. However, the images show that the 0.5TC@ZIF-8 and 0.5TC@ZIF-8@PL samples appeared to be clustered. The size measurement by NTA yielded a smaller particle size for 0.5TC@ZIF-8 and 0.5TC@ZIF-8@PL compared to the size range in the SEM micrographs. This discrepancy could be due to the fundamental differences between the two measurement techniques. In Figure 2C,D, the 0.5TC@ZIF-8 and 0.5TC@ZIF-8@PL samples appear as aggregates composed of multiple nanoparticles. This aggregation likely occurred during drying of the nanoparticles and does not represent the primary particle size in the suspension. Moreover, in NTA, the measurements were taken in suspension at different points (the sample is moved in a syringe after measurement), while SEM images represent only a small fraction of the sample. SEM images showed a variation in the size of nanoparticles, which was also confirmed by the wide size distribution ranges in NTA size measurements (Figure 2). It can be seen from the images that the PL coating did not affect the morphology of ZIF-8 and 0.5TC@ZIF-8 nanoparticles.

When ethanol was used as the solvent for synthesizing ZIF-8, following the same procedure as in this study, the resulting morphology differed from the typical ZIF-8 structure.^20^ The nanoparticles appeared to be less well defined compared to those synthesized with methanol. However, in the present study, the 0.5TC@ZIF-8 nanoparticles clearly display a characteristic ZIF-8 morphology, as evidenced by the SEM images.

Similarly, TEM images (Figure 3) show typical hexagonal ZIF-8 formation in both the ZIF-8 samples and the TC@ZIF-8 samples. The TEM images also showed a larger (5 μm scale bar) particle size for TC@ZIF-8 and TC@ZIF-8@PL nanoparticles. This implies that TC entrapment increases the size of the ZIF-8 nanoparticles.

**Figure 3:**
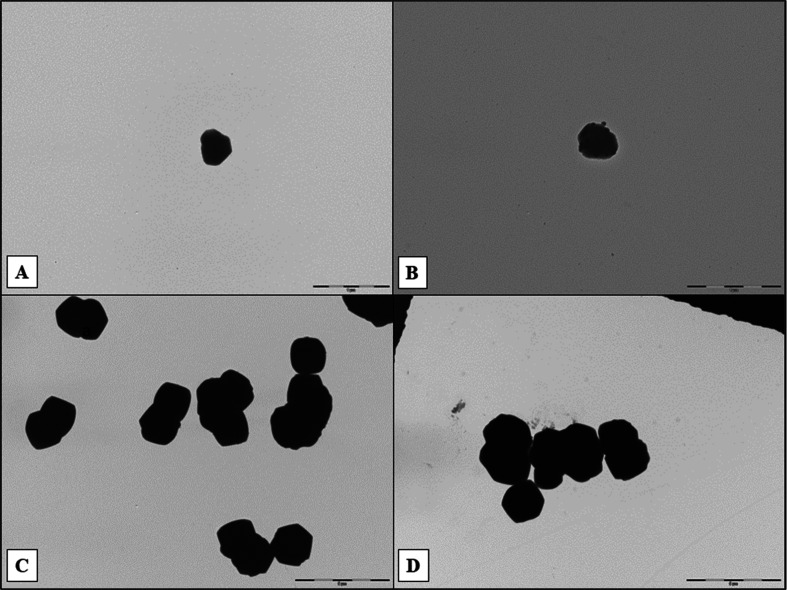
TEM images of (A) ZIF-8 in water, (B) ZIF-8 in PL, (C) 1TC@ZIF-8 in water, and (D) 1TC@ZIF-8 in PL. Scale: (A,B) 1 μm, (C,D) 5 μm.

#### XRD Analysis, Gas Adsorption Analysis, and FTIR Spectroscopy

Aliphatic alcohols have been shown to yield pure and well-developed phases of ZIF-8, although differences in relative crystallinity and crystallite size have been reported.^43^ These differences are likely due to the variations in polarity, viscosity, and size of the solvent molecule.^42,44^ Based on the XRD patterns observed in this study (Figure 4), all samples matched well with the known crystal structure of ZIF-8, confirming their purity. Characteristic peaks were observed at 2θ = 7.4, 10.4, 12.8, and 18.0, which are assigned to the (011), (002), (112), and (222) planes, respectively.^42^ The XRD pattern of TC-entrapped ZIF-8 corresponded very well to the peaks of ZIF-8, indicating that the structure remained intact after encapsulation. As the TC increased, the intensity of the peaks at 2θ = 7.4 and 12.83 initially rose and then decreased despite further increases in TC. A higher peak intensity means a higher number of atoms in the crystal. The intensity decrease in the peaks could be due to TC entrapment, yielding fewer available atoms as well as an increase in particle size. Additionally, samples coated with PL exhibited higher peak intensities compared to their uncoated counterparts (Figure 5), suggesting larger particle sizes in the coated formulations.

**Figure 4:**
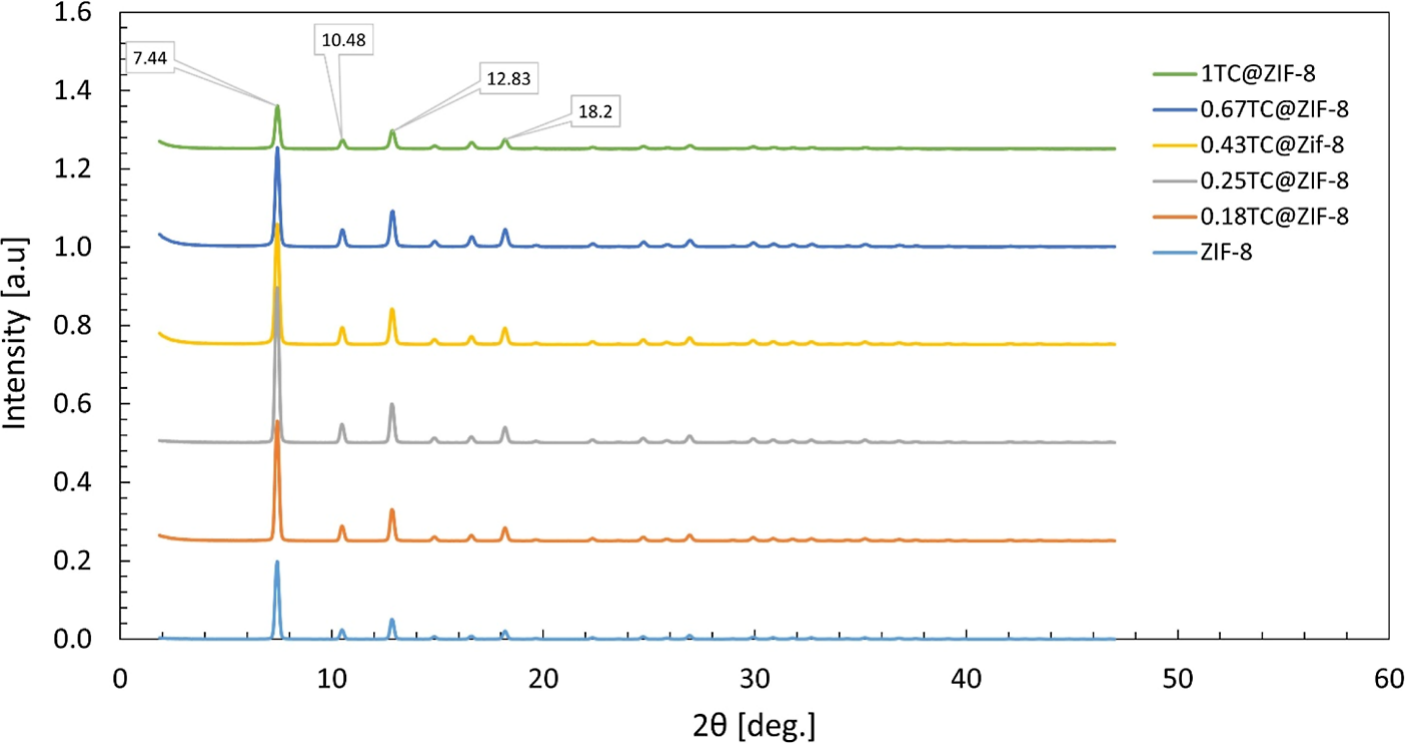
XRD pattern of ZIF-8 and TC@ZIF-8 nanoparticles.

**Figure 5:**
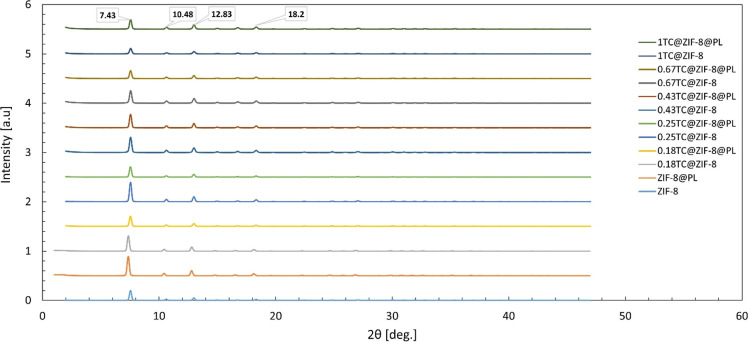
XRD pattern of ZIF-8, TC@ZIF-8 nanoparticles, and PL-coated counterparts.

The nitrogen adsorption–desorption isotherm of ZIF-8, ZIF-8@PL, 0.5TC@ZIF-8, and 0.5TC@ZIF-8@PL nanoparticles (Figure 6) showed a typical type I isotherm as reported in other studies.^20,39,45^ The amount of adsorbed nitrogen in all samples increased rapidly at low pressures, indicating the presence of micropores. ZIF-8@PL showed a slight hysteresis loop near *p*/*p*_0_ = 1, which suggests the existence of interparticle mesopores and macropores among the ZIF-8 particles.^46^ 0.5TC@ZIF-8 and 0.5TC@ZIF-8@PL nanoparticles exhibited a h4-type hysteresis loop, indicative of irregular pore structures.^47^ Similar hysteresis was observed when oregano essential oil was encapsulated into γ-cyclodextrin-based metal organic frameworks. This hysteresis could be due to pore structure change after encapsulating the essential oil, which confirms the entrapment of the drug.^47^ The reduction in nitrogen adsorption after PL coating and further reduction with TC encapsulation and PL coating supported the conclusion that the available pores are increasingly occupied by TC.

**Figure 6:**
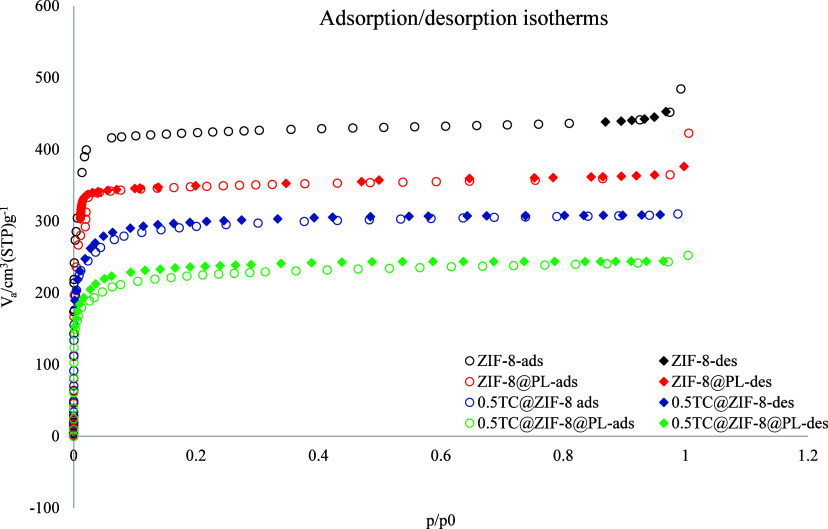
Adsorption/desorption isotherms of ZIF-8, ZIF-8@PL, 0.5TC@ZIF-8, and 0.5TC@ZIF-8@PL nanoparticles.

The specific surface area for pure ZIF-8 nanoparticles varies between 1500 and 2500 m^2^g^–1^.^48^ In this study, the BET surface area for ZIF-8 was 1932.1 m^2^g^–1^, and the decrease in surface area in TC-entrapped samples (1111.5 cm^3^g^–1^) confirms the entrapment of TC into the ZIF-8 framework (Table 4). TC-entrapped samples (0.5TC@ZIF-8) displayed a decrease in the micropore volume of 0.4674 cm^3^g^–1^ compared to ZIF-8 (0.6354 cm^3^g^–1^). Moreover, the total pore volume of ZIF-8 decreased from 0.7432 cm^3^ g^–1^ to 0.4793 cm^3^ g^–1^. All these surface characteristic changes indicate the entrapment of TC in ZIF-8 frameworks.

**Table 4:** Gas Adsorption Properties of ZIF-8 and 0.5TC@ZIF-8 and Their PL-Coated Counterparts

sample	*a*_s,BET_, m^2^ g^–1^	total pore volume, cm^3^ g^–1^	micropore volume, cm^3^ g^–1^
ZIF-8	1932.1	0.7432	0.6354
ZIF-8@PL	1422.4	0.6091	0.5376
0.5TC@ZIF-8	1111.5	0.4793	0.4674
0.5TC@ZIF-8@PL	851.65	0.384	0.3589

FTIR spectroscopy provided information about the main functional groups in the ZIF-8 and TC and TC@ZIF-8 nanoparticles (Figure 7). ZIF-8 had peaks at 420, 693, 758, 994, 1145, 1310, 1421, 1582, 2929, and 3135 cm^–1^, which agree with the previously reported data.^28^ The peaks in the bands of 2500–3500 cm^–1^ correspond to the stretching vibrations of the functional groups such as –OH, –CH_3_, and –NH–.^49^ The bands from 3200 to 3550 cm^–1^ show stretching vibrations of N–H, while the bands at 3135 and 2929 cm^–1^ are characteristics of aromatic and the aliphatic C–H stretch of the imidazole, respectively.^28^ While the peak at 1582 cm^–1^ shows axial deformation of C=N, the peak at 420 cm^–1^ corresponds to the axial deformation of Zn–N.^49^ The peaks between 1350 cm^–1^ and 1500 cm^–1^ are the result of the imidazole ring stretching.^50^ The bands at 600 to 1500 cm^–1^ are assigned to the bending vibration mode of the imidazole ring.^28^ The presence of a peak at 420 cm^–1^ confirms the coordination between Zn and 2-mim.^51^ TC had peaks at 581, 686, 744, 844, 970, 1006, 1071, 1119, 1159, 1293, 1327, 1393, 1449, 1493, 1574, 1623, 1668, 2742, 2813, 3060, and 3333 cm^–1^. It showed similar peaks to the ones reported in the literature.^52–54^ The significant peak at 3060 cm^–1^ is due to the stretching of the C–H aromatic ring, that at 2813 cm^–1^ is due to the C–H stretching of the aldehyde group, that at 1623–1700 cm^–1^ is due to the carbonyl group C=O stretching, that at 1493–1623 cm^–1^ is due to the C=C bond, and that at 1159 cm^–1^ is due to the aromatic C–H bond.^52^

**Figure 7:**
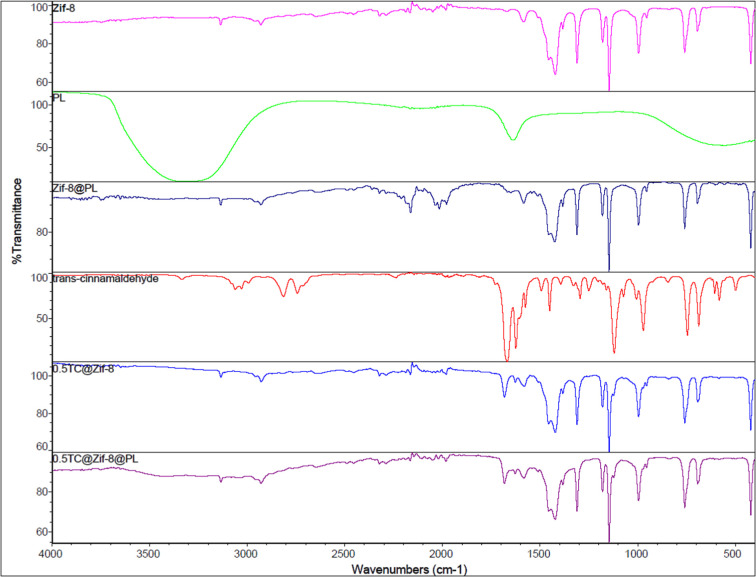
FTIR spectra of ZIF-8, TC@ZIF-8, and PL-coated counterparts.

After TC entrapment, TC@ZIF-8 had main peaks of ZIF-8 as well as additional peaks at 1626 and 1682 cm-1, which correspond to TC C=O carbonyl group stretching peaks. A minor shift in the 1582 cm^–1^ peak to 1579 cm^–1^ of ZIF-8 after TC entrapment could be due to TC and ZIF-8 interacting.

#### TC Release from ZIF-8

TC release from the ZIF-8 matrix was assessed in various media at 37 °C. In water (Figure 8), both 0.5TC@ZIF-8 and 0.5TC@ZIF-8@PL samples exhibited a slow release, reaching approximately 4.5% and 3.5%, respectively, an expected outcome given TC’s poor solubility in water.

**Figure 8:**
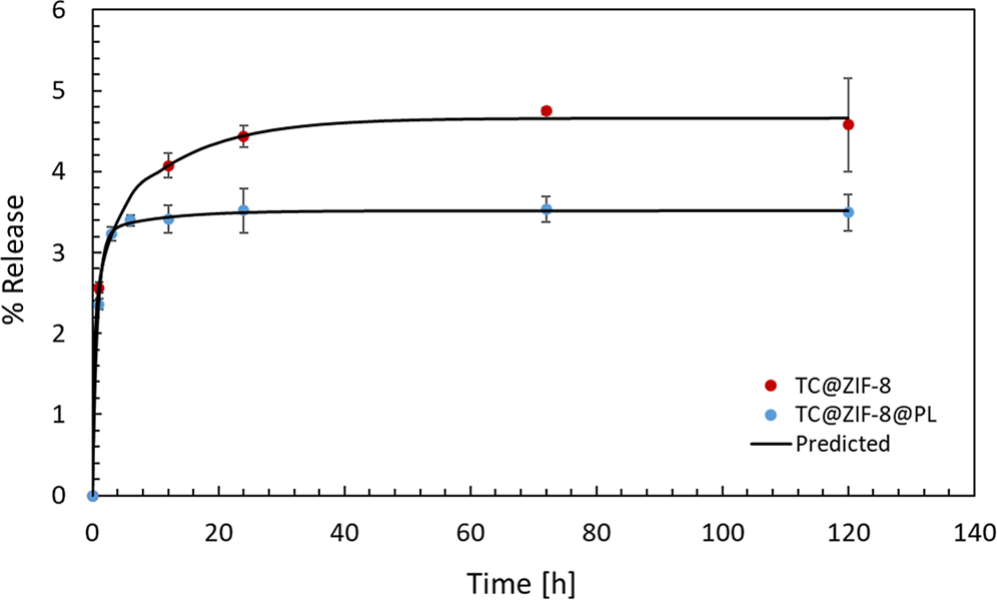
TC release from 0.5TC@ZIF-8 and 0.5TC@ZIF-8@PL nanoparticles in water.

After 24 h, the release of TC was almost constant for both samples. In contrast, when 0.5TC@ZIF-8 nanoparticles were dispersed in ethanol and methanol (Figure 9), a sustained release pattern was observed. In ethanol, the release reached about 55% within 12 h and remained stable for nearly 120 h. Similarly, in methanol, the release rose to 50% in 12 h and continued to increase, reaching 60% after 72 h.

**Figure 9:**
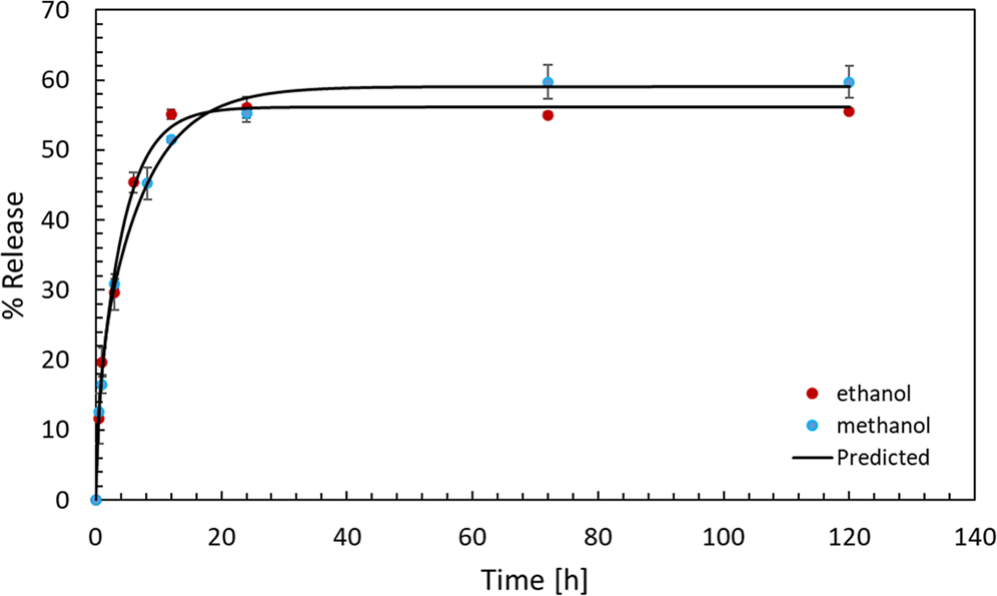
TC release from 0.5TC@ZIF-8 nanoparticles in ethanol and methanol.

The %TC release from ZIF-8 nanoparticles in water, ethanol, and methanol follows a biexponential model:

6 $$R\left(t\right)=R_{0}\left(1-\exp\left(-kt\right)\right)+R_{1}\left(1-\exp\left(-k_{2}t\right)\right)$$

where *R*(*t*) is the percentage release at time *t*, *R*_0_ and *R*_1_ are the maximum releases from two different burst phases, and *k*_1_ and *k*_2_ are the rate constants for the respective burst phases. This model accounts for an initial fast release, followed by a slower release phase, contributing to the overall burst release kinetics (Table 5). The initial fast release in water, methanol, and ethanol suggests that TC molecules were primarily located on the surface of ZIF-8 crystals, and the following slower release was due to diffusion. Since TC has poor water solubility, the % release rate was very low. On the other hand, TC is soluble in ethanol and methanol and hence the higher release.

**Table 5:** % TC Release Equations Obtained from the Biexponential Model When TC Is Released from ZIF-8 in Water, Ethanol, and Methanol

nanoparticle/media	equation	*R* ^2^
TC@ZIF-8 in water	3.0492(1 – exp(−1.6019*t*)) + 1.6105(1 – exp(−0.0837*t*))	0.99
TC@ZIF-8@PL in water	3.2392(1 – exp(−1.2696*t*)) + 0.2818(1 – exp(−0.1056*t*))	0.99
TC@ZIF-8 in ethanol	8.0134(1 – exp(−3.8492*t*)) + 48.0581(1 – exp(−0.2353*t*))	0.99
TC@ZIF-8 methanol	16.3031(1 – exp(−1.4525*t*)) + 42.4764(1 – exp(−0.2353*t*))	0.99

When 0.5TC@ZIF-8 nanoparticles were dispersed in PBS (Figure 10), a burst release of TC was observed within the first hour, reaching a peak of 23%, followed by a gradual decline in the amount released over time. To enhance the release profile, ethanol and methanol were added to PBS. A similar burst release trend was seen in PBS/MeOH mixtures (3%, 5%, and 10% v/v), but the overall TC release improved with a higher methanol content. Maximum release values reached 28.1%, 28.9%, and 29.7% for 3%, 5%, and 10% MeOH mixtures, respectively. In contrast, incorporating 20% ethanol into PBS led to a notably improved release profile—24.9% release at 30 min, with a sustained increase reaching 50% over 72 h.

**Figure 10:**
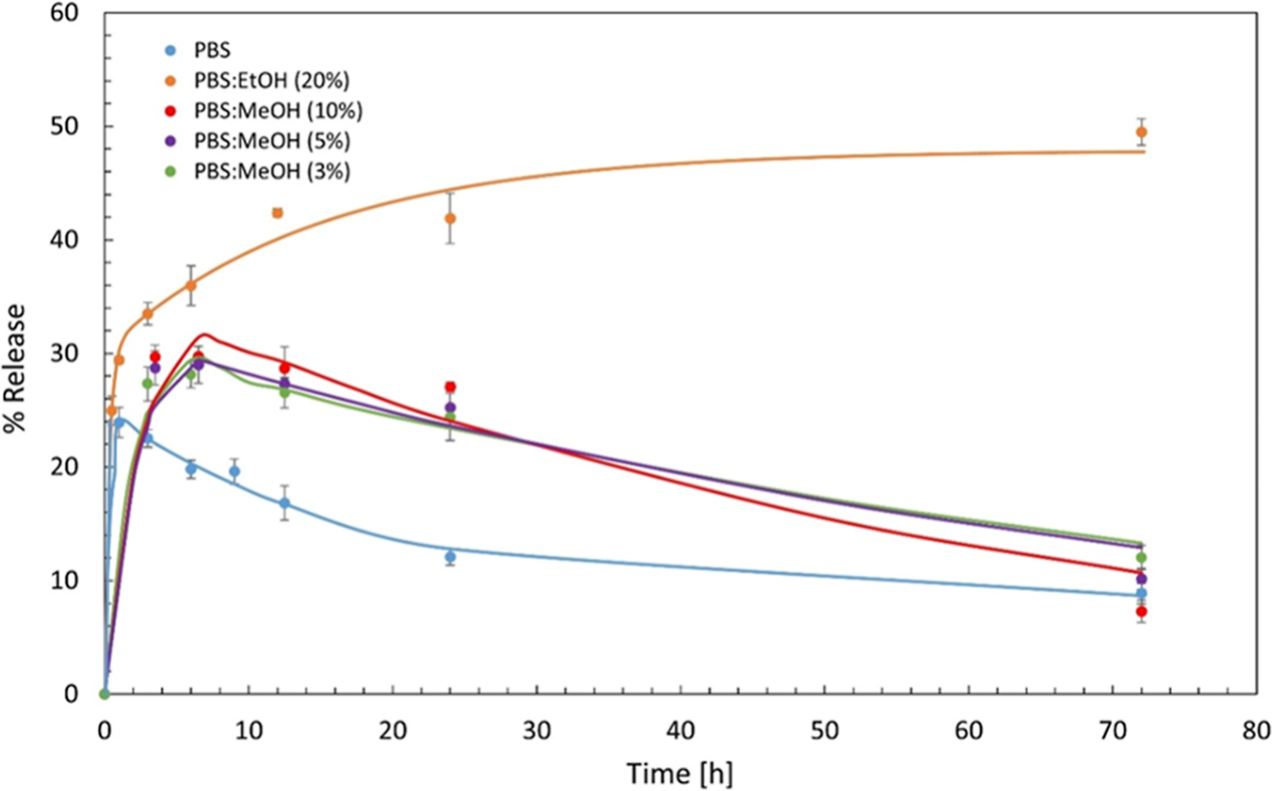
Release profile of TC from the ZIF-8 matrix in PBS and PBS/MeOH and PBS/EtOH mixtures.

ZIF-8 has been investigated extensively as a pH-responsive drug carrier. For example, a study loaded anticancer drug doxorubicin into ZIF-8 and evaluated its release in PBS across a pH range of 5.0 to pH 7.4.^19^ They found that DOX release was minimal, less than 1% at pH levels of 6.5 or higher. Another study found that curcumin released from the ZIF-8 matrix with an 88% cumulative release in an acidic medium (pH 5), whereas it was 28% in a physiological medium (pH 7.4) over a period of 72 h.^39^

A study investigated the effect of phosphate anions, both alone and in combination with lactic acid, on the degradation of ZIF-8 microcrystals.^55^ When ZIF-8 was incubated in water (pH 5.5), PBS (pH 7.4), and 0.9% saline (pH 6.3, phosphate-free), ZIF-8 showed the highest degradation in PBS, despite its higher pH, and the emergence of new crystals was observed with SEM within 2 h of incubation. In contrast, no structural damage was observed in saline after 24 h, and only minimal degradation occurred in water over time. Interestingly, even when PBS and saline were acidified using lactic acid, ZIF-8 still degraded more rapidly in PBS at pH 7.4 than in saline at pH 5.0. Acidifying PBS to pH 5.0 with lactic acid further accelerated degradation. The authors also noted that the degradation rate of ZIF-8 in the presence of phosphate anions eventually slowed, likely due to the formation of a protective zinc phosphate layer on the surface of the ZIF-8 polycrystalline aggregates.

It was found that ZIF-8 rapidly decomposed in 10 mM PBS, forming insoluble zinc phosphate particles, and the smaller ZIF-8 particles degraded faster.^56^ In this study, a decrease in TC release over time following an initial burst was observed in PBS (Figure 10), unlike the release behavior in water (Figure 8). This could be explained by the formation of zinc phosphate structures that either trap TC or create a barrier around the TC@ZIF-8 particles, slowing their release. It was also reported that the pores of ZIF-8 were hydrophobic.^57^ Therefore, only organic molecules, such as alcohol, THF, and hydrocarbons, were adsorbed in their adsorption studies. Zinc phosphate is not soluble in water, so the presence of ethanol in PBS assists in overcoming this issue by penetrating the pores easily due to its organic nature and small molecule size.

The % TC release in this study followed a biexponential model for PBS and PBS–methanol mixtures with a burst release phase, followed by an exponential decay phase. However, it followed an initial fast release phase, followed by a slower release phase, both contributing to the overall release for the PBS/EtOH mixture, like when TC was released from TC@ZIF-8 in water, ethanol, and methanol. The data were fitted (Table 6) to a combined model of a burst release phase and an exponential decay phase:

7 $$burst\;release\;phase:,R_{1}\left(t\right)=R_{0}\left(1-\exp\left(-kt\right)\right)$$

8 $$exponential\;decay\;phase:,R_{2}\left(t\right)=R_{1}\exp\left(-k_{2}\left(t-t_{c}\right)\right)$$

where *R*_1_(*t*) is the percentage release at time *t* during the burst phase, *R*_0_ is the maximum release during the burst phase, and *k* is the rate constant for the burst phase. *R*_2_(*t*) is the percentage released at time *t* during the exponential decrease phase, *R*_1_ is the percentage release remaining after the burst phase, *k*_2_ is the rate constant for the exponential decrease, and *t*_c_ is the transition time from the burst phase to the exponential decrease phase. Combining eqs 7 and 8

9 $$combined\;model:,R\left(t\right)\{\begin{array}{l} R_{0}\left(1-\exp\left(-kt\right)\right)\\ R_{1}\left(t_{c}\right)+R_{1}\exp\left(-k_{2}\left(t-t_{c}\right)\right) \end{array}$$

**Table 6:** % TC Release Equations Obtained from the Biexponential Model When TC Was Released from ZIF-8 in PBS and PBS/EtOH and PBS/MeOH Mixtures

release medium	equation		*R* ^2^
PBS	BRP^a^	21.401(1 – exp(−0.0549*t*))	0.98
	EDP^b^	8.348 + 16.6448 exp(−0.0549*t*))	0.99
PBS/MeOH (10%)	BRP	33.5117(1 – exp(−0.4246*t*))	0.95
	EDP	32.2998 exp(−0.0169(*t* – *t*_c_))	0.92
PBS/MeOH-5%	BRP	32.6161(1 – exp(−0.4280*t*))	0.98
	EDP	30.4738 exp(−0.0143(*t* – *t*_c_))	0.96
PBS/MeOH-3%	BRP	30.4721(1 – exp(−0.564*t*))	0.95
	EDP	29.4362 exp(−0.0126(*t* – *t*_c_))	0.96
PBS/EtOH-20%	30.2279(1 – exp(−3.1864*t*)) + 17.6543(1 – exp(−0.068*t*))		0.99

a BRP = burst release phase.

b EDP = exponential decrease phase.

Table 7 shows the pH values of the release media used in this study, and the results align with other findings.^55^ PBS, despite having a pH higher than that of water, resulted in greater TC release. Although both water and methanol exhibited similar acidic pH levels, they produced different % TC release amounts. The molecular weight of methanol (32.042) is smaller than that of ethanol (46.07 g/mol).^58,59^ This smaller molecular size may allow methanol to penetrate the ZIF-8 pores more effectively, facilitating higher TC release. These findings suggest that TC release in PBS was a combination of surface desorption and degradation of the ZIF-8 matrix due to phosphate anions, rather than pH-responsive degradation.

**Table 7:** pH Values of Different Release Media

release medium	pH^a^
water	6.96 ± 0.02
ethanol	7.24 ± 0.06
methanol	6.98 ± 0.10
PBS	7.40 ± 0.00
PBS/MeOH (5%)	7.50 ± 0.02
PBS/EtOH (20%)	7.87 ± 0.02

a Values given are averages of three replicate samples ± standard deviations.

### Antibacterial Activity

In this study, TC demonstrated antibacterial activity against *E. coli* MG1655 at a concentration of 0.25 mg/mL (Table 8). Similarly, an inhibitory concentration of 0.14 mg/mL was reported against *E. coli* CVCC1567,^60^ while another study^32^ found a concentration of 0.5 mg/mL to be effective against *E. coli* O157:H7. The antibacterial efficacy of essential oils like TC can vary based on several factors, including the plant source, harvest timing, extraction method,^61^ and the specific bacterial strains tested. TC’s antimicrobial mechanism is primarily attributed to its ability to disrupt the bacterial cell wall by disturbing the balance between lipopolysaccharides (LPS) and phospholipids.^60^

**Table 8:** MICs and MBCs of TC, PL, ZIF-8, and TC@ZIF-8 against *E. coli* MG1655 after 24 h of Exposure

compound	MIC^a^, mg/mL	MBC^b^, mg/mL
TC	0.25	1
PL	>0.25^c^	n/a
ZIF-8	3	>10^c^
ZIF-8@PL	5	>10^c^
0.5TC@ZIF-8	2.5	4
0.5TC@ZIF-8@PL	4	7
0.5TC@ZIF-8 (in PBS)	1.75	n/a
0.5TC@ZIF-8 (in PBS/MEOH)	1.25	2

a Values are the lowest concentration of antimicrobial compounds for which a ≤OD630NEG was observed after 24 h of incubation at 37 °C in TSB.

b Values are the lowest antimicrobial concentration that provided 3 log reduction from the initial bacterial population.

c Values preceded by a higher than (>) means that tested concentrations were not sufficient to determine the MIC and MBC values. n/a MBC test was not performed.

PL did not show any antibacterial activity at the tested concentration. In contrast, all other tested materials demonstrated antibacterial activity. The antibacterial effect of ZIF-8 comes from metal ions in the framework. Zn^2+^ ions can show antimicrobial activity like ZnO nanoparticles when they are partially dissolved in water. This action is linked to their ability to interrupt the cell membrane by electrostatic interactions (zinc ions) or generate reactive oxygen species (ROS) as well as bind the proteins and DNA and modify the gene expression.^62^ In this study, ZIF-8 and ZIF-8@PL showed inhibitory activity at 3 and 5 mg/mL, respectively. However, neither of them showed bactericidal activity at the tested concentrations, as both would require concentrations higher than 10 mg/mL.

Various antibacterial agents have been entrapped into the ZIF-8 matrix, including ciprofloxacin,^63^ physcion,^64^ and vancomycin^65^ to enhance antibacterial performance. In this study, when 0.5TC@ZIF-8 nanoparticles dispersed in water, they had an inhibitory effect on *E. coli* at a concentration of 2.5 mg/mL (Figure 11, top); however, when these nanoparticles were coated with PL (0.5TC@ZIF-8@PL, Figure 11, bottom), the inhibitory concentration increased to 4 mg/mL and the minimum bactericidal concentration rose from 4 mg/mL to 7 mg/mL.

**Figure 11:**
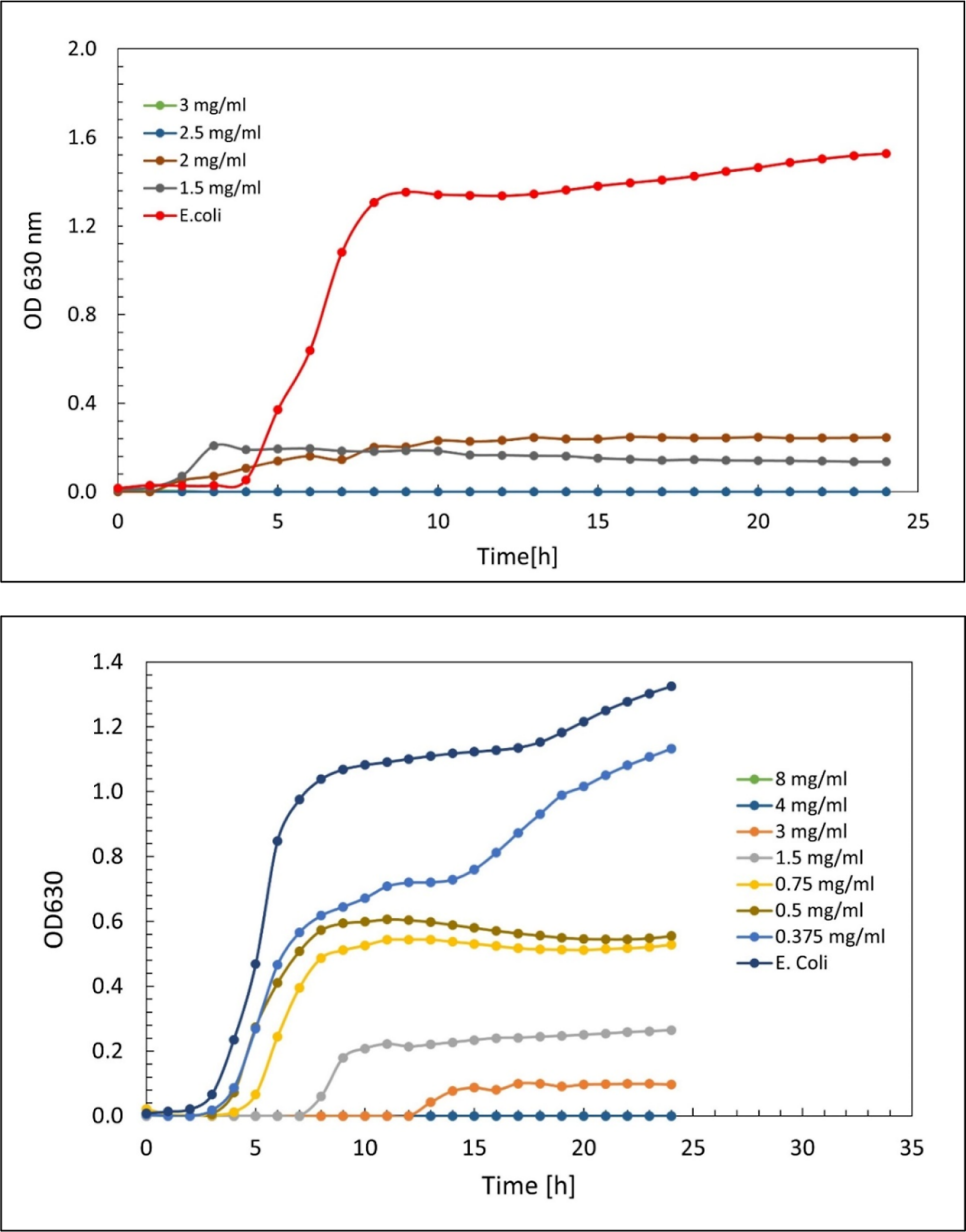
Growth curve of *E. coli* MG1655 with 24 h of treatment with 0.5TC@ZIF-8 in water (top) and 0.5TC@ZIF-8@PL in water (bottom).

As shown in Figure 11, 0.5TC@ZIF-8 exhibited dose-dependent inhibition of *E. coli* MG1655 growth, with inhibition observed at 2.5 mg/mL and above, whereas partial inhibition occurred at lower concentrations. In contrast, 0.5TC@ZIF-8@PL required higher concentrations to achieve growth inhibition, with complete suppression observed only at ≥4 mg/mL, while subinhibitory concentrations (0.375–1.5 mg/mL) allowed bacterial growth with a delayed lag phase compared to the untreated control. The lag phase is a temporary period of nonreplication. It is commonly described as the preparation phase that allows the bacteria to adapt to the new environment.^66^

These results suggest that while PL coating provides stability benefits, it reduces the antibacterial efficiency of TC@ZIF-8, likely due to altered release dynamics of encapsulated TC. The required inhibitory concentration can be significantly reduced simply by modifying the dispersion medium. As shown in Figure 12, 0.5TC@ZIF-8 in PBS inhibited *E. coli* growth in a concentration-dependent manner. Complete inhibition was observed at concentrations of ≥1.75 mg/mL, while partial suppression occurred at 1.25 mg/mL, where cells exhibited delayed but detectable growth.

**Figure 12:**
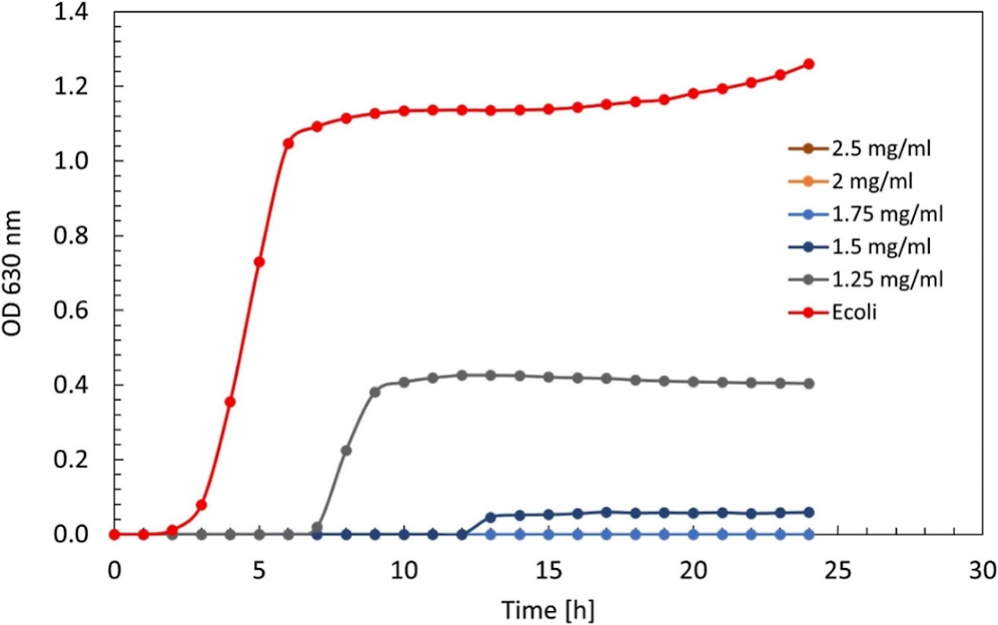
Growth curve of *E. coli* MG1655 with 24 h of treatment with 0.5TC@ZIF-8 in PBS.

This effect was even more pronounced when 5% methanol was added to the PBS (Figure 13), lowering the MIC further to 1.25 mg/mL. Additionally, not only did the MIC decrease, but also the minimum bactericidal concentration (MBC) of 0.5TC@ZIF-8 was reduced by half when using the PBS/MeOH (5%) mixture instead of water, highlighting the importance of medium composition in optimizing antimicrobial performance.

**Figure 13:**
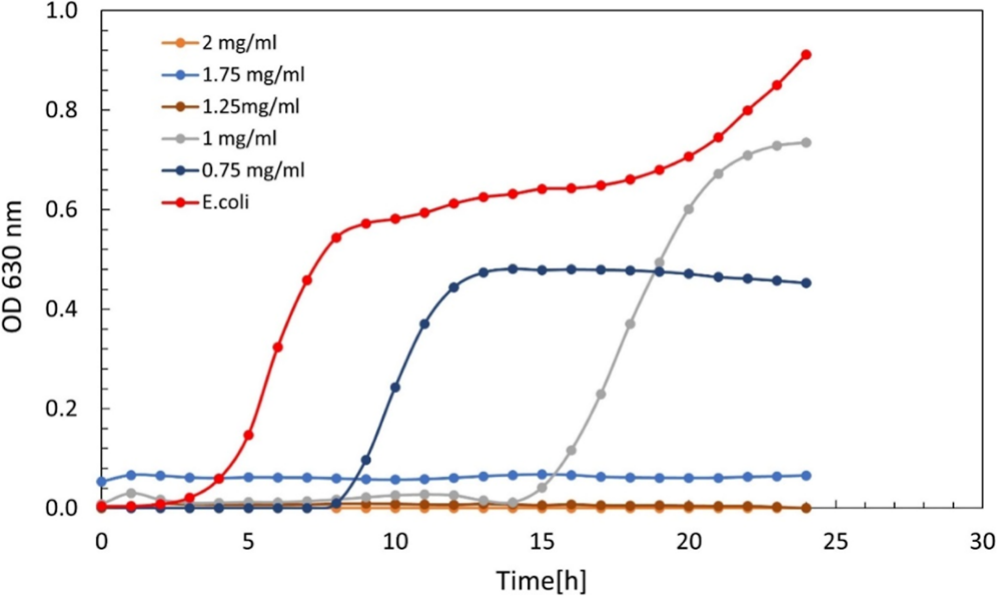
Growth curve of *E. coli* MG1655 with 24 h of treatment with 0.5TC@ZIF-8 in PBS/MeOH.

These findings indicated that while ZIF-8 can serve as a carrier to deliver TC with improved efficacy over ZIF-8 alone, both surface modifications and dispersion conditions significantly impact antimicrobial outcomes.

## Conclusions

This study aimed to develop and then characterize *trans*-cinnamaldehyde-loaded ZIF-8 nanoparticles with antibacterial properties via a one-pot method. UV–vis spectra confirmed the presence of TC in the synthesized nanoparticles (TC@ZIF-8). The best TC to zinc + 2-mim ratio was 1:2, which yielded the highest entrapment efficiency. The size measurements showed that ZIF-8 nanoparticles were in the range of 100–200 nm and in agreement with entrapment efficiency values, although PL coating increased the particle size beyond the 300 nm range.

SEM and TEM images confirmed the characteristic morphology of ZIF-8 in the nanoparticles, as well as TC-entrapped ZIF-8, validating the observation that TC entrapment did not change the morphology of ZIF-8 nanoparticles. Gas adsorption analysis yielded a high BET surface area, which decreased with TC entrapment. FTIR spectra exhibited distinctive peaks of ZIF-8 and TC in the TC@ZIF-8 nanoparticles, and both methods further confirm the successful method for nanoparticle synthesis.

TC release studies revealed that 0.5TC@ZIF-8 nanoparticles exhibited a burst release in PBS buffer but a more controlled, steady release in water and alcohols. All synthesized ZIF-8 nanoparticles showed some degree of antibacterial activity. An interesting finding was that the antibacterial activity of 0.5TC@ZIF-8 nanoparticles could be increased by changing the dispersion media.

## Supplementary Material



## Notes

Schematic of the laboratory procedure used to synthesize TC-entrapped ZIF-8 (TC@ZIF-8) outlining key steps including precursor mixing, entrapment of TC during ZIF-8 formation, purification, and drying, designed to produce nanoparticles for antibacterial applications in food safety (PDF)
