# Supplementary material for: Development and Characterization of trans-Cinnamaldehyde-Entrapped Zeolitic Imidazole Framework‑8 as an Antibacterial Agent for Food Safety Applications
Source: ACS Omega. 2025 Sep 18;10(38):43855–70. doi: 10.1021/acsomega.5c04494 (PMC12489854; doi:10.1021/acsomega.5c04494)
Supplement: Supplementary file 1 [file ao5c04494_si_001.pdf]

Supplementary materials

Manuscript title: Development and Characterization of trans-Cinnamaldehyde Entrapped Zeolitic Imidazole Framework-8 as an Antibacterial Agent for Food Safety Applications

Authors: Zeynep Sevimli Yurttas[1], Rosana G. Moreira[2], Elena Castell-Perez[2]*

[1]Research Assistant, [2]Professor, * Corresponing author
Department of Biological and Agricultural Engineering, Texas A&M University,
College Station, Texas, USA

*311 Scoates Hall
Texas A&M University
College Station, TX 77843-2117
(979) 314-8249
E-mail address: Elena.Castell-Perez@ag.tamu.edu

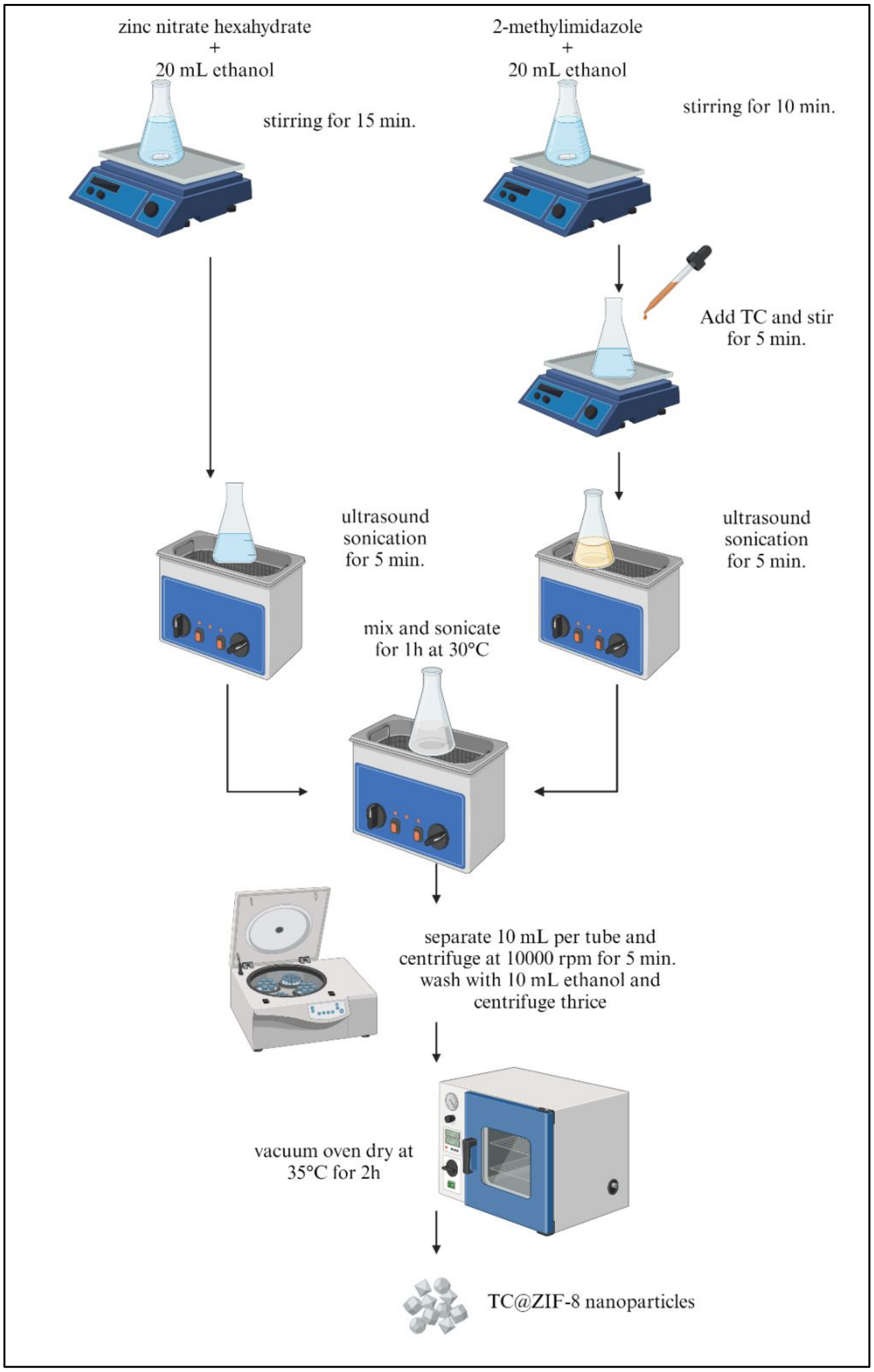

Figure S1. Schematic illustration of TC@ZIF-8 synthesis procedure. (Created with BioRender.com)
